# School health education program in Pakistan (SHEPP)—a threefold health education feasibility trial in schoolchildren from a lower-middle-income country

**DOI:** 10.1186/s40814-020-00625-x

**Published:** 2020-06-08

**Authors:** Aysha Almas, Romaina Iqbal, Sania Sabir, Abdul Ghani, Khawar Kazmi

**Affiliations:** 1 Department of Medicine, Aga Khan University, Karachi, Pakistan; 2 Department of Medicine and Community Health Sciences, Aga Khan University, Karachi, Pakistan; 3 National Institute of Cardiovascular Diseases, Karachi, Pakistan

**Keywords:** Physical activity, Diet, Health education, Adolescents, Cardiovascular, School

## Abstract

**Background:**

The school environment plays an essential role in promoting health education and physical activity for children and adolescence, and they are more likely to adapt it into their adulthood. School health education program has been endorsed and emphasized by the World Health Organization has not been implemented in true spirit in Pakistan yet. We aim to test feasibility of threefold health education program in children and its potential efficacy on physical activity and diet and cardiometabolic risk factors by including BP, BMI, and waist circumference.

**Methods:**

It is a parallel-group feasibility intervention trial. It is being conducted in two schools from lower to middle-income areas, at different locations but having the same school curriculum under the Aga Khan Education Service, Pakistan (AKESP). All children aged 9-11 years enrolled from the schools mentioned above were included. Children with any physical disability were excluded. One school received threefold intervention (focused on children, parents, and teachers) of school health education program in Pakistan (SHEPP) while the other school continued routine activity. Intervention of SHEPP is directed towards educating children, parents, and teachers about healthy behaviors. Children will receive interactive educational sessions and specially designed physical activity sessions. A 3-h health education session focusing on same healthy behaviors as for children will be conducted for both parents and teachers. Primary outcome is feasibility of SHEPP in terms of recruitment, retention, and treatment fidelity. Secondary outcomes are physical activity levels, dietary intake (of fruits, vegetable), and cardiometabolic risk factors (blood pressure, BMI, and waist circumference (WC)). The total number of children recruited were 982 (82.5 %); 505 from school A and 477 from school B and 496 (50.5) were boys.

**Conclusion:**

SHEPP is a unique health education program for children as it focuses on children while involving the parents and teachers in the behavior change process. If found feasible and demonstrating potential efficacy on physical activity, dietary behaviors, and cardiometabolic parameters, we will be able to replicate this on a larger scale in public sector schools also.

**Trial registration:**

NCT03303287

## Background

Cardiovascular diseases (CVD) top the list of the five leading causes of death globally [1]. CVD accounts for a third of the deaths in Pakistan [2]. In a lower-middle-income country like Pakistan, where an organized health infrastructure is still in infancy [3], the only cost-effective way to tackle this rising burden of CVD could be by providing health education to children and adolescents at a mass level about healthy lifestyle [4]. High childhood and adolescent BMI are associated with an increased risk of cardiovascular disease in adulthood [5]. Thirteen percent of children from Karachi, Pakistan, have been observed to be obese (higher BMI) and 21% had greater abdominal obesity.

Lack of adequate physical activity (PA) in children is a known risk factor of obesity. The recommended guideline for PA among children and youth (WHO, [6]) is that children and youth aged 5–17 should accumulate at least 60 min of moderate- to vigorous-intensity physical activity daily. But in a lower-middle-income country, like Pakistan, only 7% of the girls and 30% of the boys aged 13–14 years do the recommended physical activity of 1 h per day. About 66% of the children attending school reported that they did not participate in organized sport within school [7]. The reason for this inadequate physical activity include lack of spaces available for physical activity in school and out of school, cultural barriers for girls to do physical activity outside their house, less designated time for physical activity in school, and out of school by teachers and parents respectively (due to lack of awareness about benefits of physical activity) [8]. Approximately 85% of the students had a predominantly sedentary lifestyle, due to tuitions, television viewing, or internet surfing or indoor games like play stations in affluent schools of Karachi [9]. Additionally, a previous study from Pakistan has reported that unhealthy food, especially sugar-sweetened beverage consumption, and insufficient physical activity were associated with overweight and obesity [10]. Prevalence of smoking was reported to be 14% in adolescents aged 5-15 years from Pakistan [11, 12]. Additionally, the overall prevalence of smokeless tobacco and/or betel quid use is 42.6% in a study conducted on secondary school adolescents (*n* = 2140) [13].

Studies have been done to incorporate healthy behaviors in children like the good behavior game (GBG) to prevent the use of tobacco [14, 15]. The PAX version of the GBG (PAX GBG, developed by the Paxis Institute in the USA) was implemented in Estonia for the first time in 2014 by the Estonian National Institute for Health Development (NIHD) [16]. It is a classroom-based game where students are reinforced for their mutual success in withholding inappropriate behavior. As the preschool era in the life of a child plays an important role in developing healthy or unhealthy living patterns in adult life, designing of interventions to educate these adolescents about a healthy lifestyle is of prime importance in a lower-middle-income country like Pakistan.

Dietary or physical activity behavior produces a significant and clinically meaningful reduction in body mass index status of children and adolescents in preventing obesity [17, 18]. Additionally, school health interventions are effective in preventing smoking, and bullying [19]. While school health education program has been endorsed and emphasized by the WHO, it has not been implemented in true spirit in Pakistan. The health education that is provided is rather embedded within the course work and is not comprehended and does not add to behavioral change by children in the way it should. The main reason behind this lack of implementation of such programs is that public health education in Pakistan does not meet current national challenges which could regulate education, harmonize global standards to local context, or develop relevant career pathways [20]. Secondly, there is lack of rigorous evaluations of both educational content and programs in schools [21].

Theory of planned behavior is one behavior change theory that has demonstrated usefulness in studies of health-related behavior [22]. This suggests that educational strategies aimed at increasing children’s motivation remain an important strategy to promote physical activity. A review on the contextual influences on physical activity and eating habits of the community level reports that successful community-based health promotion strategies should consist of multilevel-multicomponent interventions on different levels of environments [23]. Such interventions in promoting a healthy lifestyle in children are targeting children, parents, and teachers simultaneously by introducing learning sessions and materials for a healthy lifestyle is useful [24, 25]. This threefold intervention including children, parents, and teachers is necessary as children learn behaviors from both school and home environment and if there is a disagreement between the two, the child might face conflicting behaviors that are hard to follow. We have previously shown the feasibility of a school-based physical activity program in a public sector girl’s school of urban Pakistan showing a favorable trend in BP and BMI at follow-up. However, the study did not include any health education intervention targeting children, teachers, or parents.

The aims of this study are the following:

1. To test feasibility of threefold health education program (on children, teachers, and parents) in school children.
2. To test the potential efficacy of such a program on physical activity, diet, and cardiometabolic risk factors including BP, BMI, and waist circumference.

## Methods

### Study setting

**Fig. 1:**
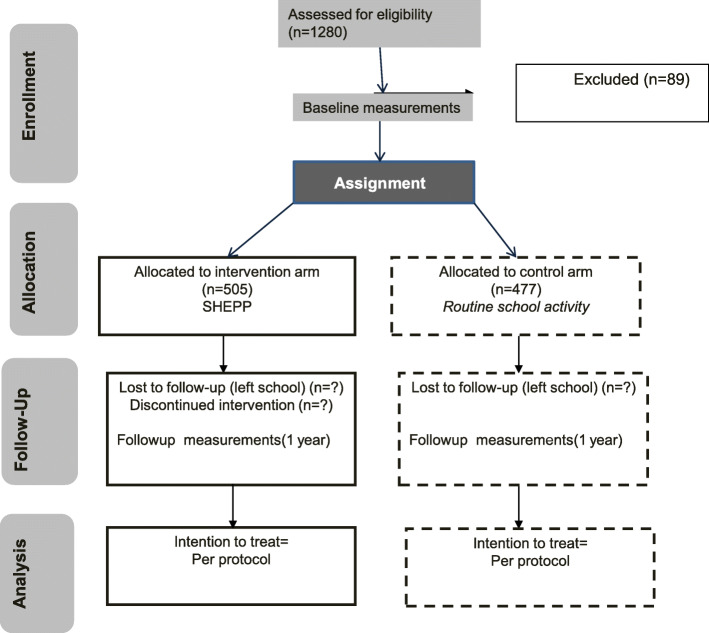
Study flow at baseline and follow-up at 12 months in the SHEPP

It is a parallel-group feasibility intervention trial. The justification for a feasibility design is that this is the first study to incorporate health education, physical activity intervention in children involving parents, teachers, and children. Secondly, feasibility studies are relied on to produce a set of findings that help determine whether an intervention should be recommended for future efficacy testing or not [26]. This feasibility intervention design to test SHEPP is therefore justified. It is being conducted in two schools located in lower to middle-income class, at different locations but having the same school curriculum under the Aga Khan Education Service, Pakistan (AKESP). Each school has approximately 2000 students, both boys and girls. Each school comprises of 10 levels of classes with 3-4 sections each. These 10 levels of classes are similar to the Cambridge O Level (but the curriculum is locally adapted), where level of education increases with each increasing class from 1 to 10. Sections refer to groups of children sitting in separate rooms but at the same level of education. The study is expected to complete in 18 to 20 months. The study flow is shown in Fig. 1. All children aged 9-11 years enrolled from the abovementioned schools were included. The rationale for this age limit is that in this period of 9-11 years age, physical activity starts to decline mainly in girls [27]. Those suffering from any physical disability were excluded from this feasibility study. If found feasible, specific physical activity for disable children by using additional aids might be useful in future studies. These children however will attend the health education teaching sessions according to the principles of ethical justice.

### Interventions

**Fig. 2:**
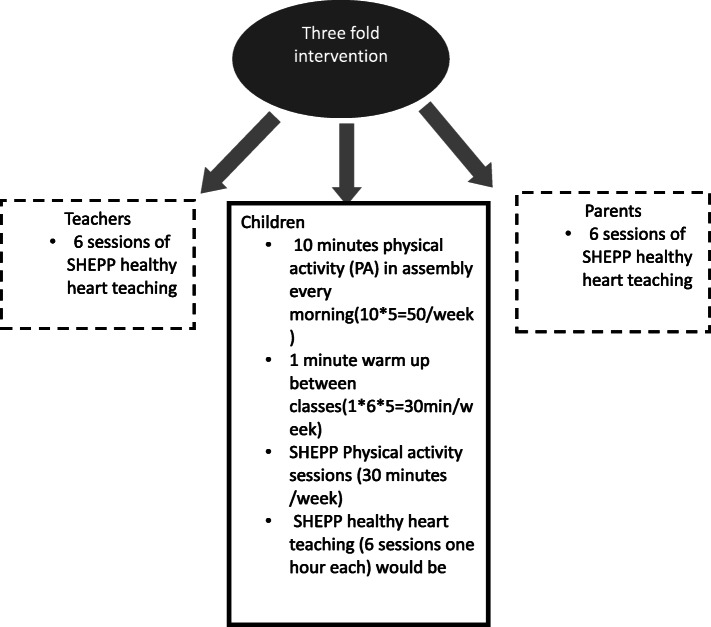
School health education program in Pakistan (SHEPP)

Intervention is the school health education program in Pakistan (SHEPP). SHEPP is directed towards children, parents, and teachers (Fig. 2). The basis of designing such intervention is to overcome cultural, socioeconomic barriers in performing physical activity [28]. The primary focus will be children. Intervention will be conducted within school premises over 10 months in one school while routine activities will be carried out in the control school. The 10 months cutoff has been chosen as benefits on cardiovascular risk factors are more pronounced at 10-month follow-up [29].

### SHEPP-children

The SHEPP focusing on children comprises of physical activity sessions and healthy heart teaching.

#### SHEPP-physical activity sessions

These sessions in schools will comprise of total 140 min/week comprising of (a) 30 min twice a week delivered by teachers trained by physical trainers. It will be based on principle of warm-up, moderate to vigorous physical activity (games), and cool down [30] (30 × 2 = 60 min/week). This has been previously tested [30]. At least 12 different SHEPP-physical activity sessions have been designed for the participants. These were designed in coordination with the physical activity trainers of school. Every 30 min physical activity sessions comprise of 10-15 min structured exercise and 15-20 min games. (b) 10 min physical activity daily during assembly (10 × 5 = 50 min/week)*.* (c) 1 min between class physical activity (1 × 6 × 5 = 30 min/week) (Table 1: SHEPP physical activity sessions outline).

**Table 1:** SHEPP-physical activity sessions

	Structured exercise	Games
Duration	10-15 min	15-20 min
*Level I* (*basic*)		
Session 1	Yoga	Ball with music or pass the ball
Session 2	Warm-up jogging	Race with ball
Session 3	Run and jump	Throwing stations
Session 4	Let us warm-up	Obstacle race
Session 5	Hopping race	Trees and squirrels
Session 6	Partner race	Couch potato tag
*Level II* (*advanced*)		
Session 1	Warm-up jogging Animal actions	Relay race
Session 2	Warm-up jogging Move and freeze	Lemon and spoon race
Session 3	Warm-up jogging	Hide and seek
Session 4	Warm-up jogging, aerobics	Running and catching (Pakram Pakrai)
Session 5	Warm-up jogging	Star war throwing game
Session 6	Warm-up jogging	Hopping race

#### SHEPP healthy heart teaching

This comprises 6 sessions of 30 min each. The sessions will be interactive using brief pictorial presentations, videos, and an activity to make it interesting for the children. In addition to healthy heart teaching additional stay clean and PAX, good behavior game will also be introduced to children. These additional sessions were identified as a “need” for the school children in preliminary discussions with teachers [31, 32]. The PAX GBG begins with creating a shared class vision displayed in the classroom for developing and maintaining a supportive classroom environment. The children will be introduced to good and bad (spleem) behaviors. Five teams will be made in each class with a team leader. A timer will be set during which children will be observed for good and spleem behaviors. The team which shows a spleem behavior will be given 1 mark. The team that has less marks at the end of the game will be the winner and will receive a prize at the end of the game. Examples of good behaviors are raising a hand when asking the teacher or doing work quietly while an example of spleem behavior is making noise or speaking without permission of the teacher [16]. Table 2 shows the outline of the SHEPP healthy heart teaching. The healthy heart teaching was based on resources from the American Heart Association (https://www.heart.org).

**Table 2:** SHEPP healthy heart teaching

	Title	Description	Activity
Session 1	A happy heart; *what makes heart happy and what makes it sad*	What is the heart? Why is it important? What is good for the heart? What is bad for the heart?	Feeling of heart by hand Counting pulse of each other Checking pulse after spot jogging Ask them to pick pictures that are good for heart and bad for heart
Session 2	The smart diet	What is smart and healthy diet? My food plate	Pictures of food items given to children and they were asked to pick the healthy ones
Session 3	Keep moving	Why physical activity is good? Why using tablet/watching TV for long is bad? How to keep moving?	Ask them to jog, and how they feel (happy or sad) Speak up on watching TV or watching tablet
Session 4	Smoking cigarette or chewing “gutka” is bad for health	Why smoking cigarette is bad? What is second-hand smoking? Is it harmful?	Ask all students to write 2 harmful effects of smoking
Session 5	Stay clean, stay healthy	Why do hand washing? Why brushing teeth is important?	Perform and demonstrate in front of class correct way of washing hands and brushing teeth
Session 6	Pax good behavior game	Make children learn good class behaviors Make note of not so good class behaviors Not point out any child on bad behavior Only point teams Appreciate with Pax action prize	Playing Pax behavior game

### SHEPP-teachers

The SHEPP for teachers is based on the same core topics as shown in Table 2; however, some of the topics were changed to make it more relevant for the teachers (as they were adults). Thirty teachers involved in teaching the respective classes as recommended by the school principal will be invited. The 3-h interactive workshop will be conducted on a weekend when teachers had come for other administrative work in school. The workshop will be delivered by trained research staff under the close supervision of the primary investigator. A teaching manual for the teachers will also be developed with a table of specifications for different sections of the workshop. The outline of the workshop is shown in Table 3. The teaching methods included (1) presentations by facilitators, (2) interactive discussion between facilitators and teachers, (3) healthy physical activity during workshop, and (4) discussion on personal stories of teachers. There will be no formal competency assessment of the teachers on their understanding of the SHEPP due to resource and time constraints, but ideally should be incorporated in future larger studies to encourage participation in the workshop a free health checkup for the teachers including blood pressure measurement, glucose monitoring, height and weight measurements (BMI calculation) will also be offered. These services will be provided by other allied health workers.

**Table 3:** SHEPP for teachers and parents

Topics	Subtopics	Time duration
1. The heart attack!	What does the heart do and how? Understand how blockage occurs in coronary arteries and why you should care. What is heart attack or disease? Risk factors for CAD	30 min
2.What is healthy diet	What is meant by a healthy diet? My food plates Daily calorie requirement Hints for healthy eating	30 min
3.Keep moving and stay active	Why moving and staying active is important? Sitting and using mobile phones/computer/watching TV is bad What are the recommendations and how to move?	30 min
4. Why smoking, “gutka, shisha and chaalia” is bad	Why smoking is harmful? Shisha, gutka, and chalia Ways to quit smoking	30 min
5. Wash your hands always!	Proper hand hygiene Dental hygiene	30 min
6. Stay calm, stay away from anger and stress	Anger and stress How to be less stressed and less angry	30 min

### SHEPP-parents

The SHEPP for parents is based on the same outline as for teachers (Table 3). Parents will be invited twice on a weekend to attend a 3-h healthy heart teaching. This will be done by the team of trained staff and a physician. To encourage in the workshop a free health checkup for the parents including blood pressure measurement, glucose monitoring, height and weight measurements (BMI calculation) will also be offered

### SHEPP-routine activity in control group

The school in the control group will carry on routine physical activity in school which is a 30-min physical activity as per schedule in school. For ethical reasons (1) a workshop for teachers will be held as for SHEPP teachers in the intervention arm towards the end of the study, (2) health education posters will be placed in school classes towards the end of the study, (3) a large class format combined health tips (on physical activity and diet) will be held for all children after completion of study.

## Adherence to SHEPP

A logbook will be maintained to track SHEPP-physical activity and healthy heart sessions for children. The adherence will be measured in terms of percentage of sessions attended by the children. To improve adherence physical activity leaders will be selected from each class and will be given a badge of physical activity leaders. Additionally, after completion of the initial 12 sessions of physical activity, children will be asked “If they had any other games they wanted to play” and these will be incorporated into the SHEPP physical activity sessions. To improve adherence to intervention of SHEPP for teachers and parents, free health checkups will be conducted along with healthy heart sessions.

## Outcome measures

### Measures of feasibility

Feasibility of SHEPP in terms of recruitment, retention, and treatment fidelity will be assessed. *Recruitment* is defined as the percentage of participants enrolled out of the total participants who were invited at baseline. *Retention* is defined as the percentage of participants who will be available for follow-up at 10 months out of those recruited at baseline. *Treatment fidelity* will be defined as the proportion of planned physical activity sessions held [33]. The trial will be considered as feasible if recruitment, retention, and treatment fidelity are > 70% recruitment [34].

Measure of potential effect

Physical activity levels (in school, out of school, moderate to vigorous physical activity, and sedentary time) assessed by change in time (minutes) spent in physical activity. Dietary patterns in terms of percentage increase in fruits and vegetable serving/day, and percentage decrease in sweetened beverages and snacks/day, cardiometabolic risk factors by change in BP, BMI, and waist circumference (WC).

## Sample size

Since this is a parallel-group feasibility intervention trial in a school setting, where interventions can only be done on class level (in a group) and not individually due to ethical and administrative reasons, we included 12 classes of children from the intervention school and 12 from control school. Each class has approximately 40-45 students making a total sample size of approximately 540 participants in intervention and 540 in the control arm. This sample size is sufficient to measure feasibility outcomes of 70% recruitment, retention, and treatment fidelity. As mentioned by Thabane et al., for an expected completion rate (recruitment, retention, and treatment fidelity for this study) of 75%, the minimum required sample for the pilot study would be at least 75 participants using a 95% CI for the proportion and a margin of error (ME) of 0.05, a lower bound of this CI of 0.70 [35]. Hence, the abovementioned sample size for this study is sufficient to assess the feasibility outcomes.

## Recruitment

We aimed to recruit children from schools working under the AKESP, primarily because, all these schools have the same design of curriculum and extracurricular activities. There are 3 schools working under the AKESP and based on logistics, we decided to conduct SHEPP in 2 schools within 10-km distance from the study center. Approval was taken from the head of schools in AKESP. After these principals of both schools were approached and at least 2 detailed meetings were held to discuss the designing of the study and recruitment. List of children aged 9-11 years studying in the morning shift (in general class 2 and 3) were obtained from the principals, and 1280 students were assessed for eligibility. After this informed consent and assent forms were distributed in each class by the PI and research staff of both schools. A note was also written in their dairies so parents could understand about SHEPP. One-week time was given to parents to agree or disagree to consent. Parents were given contact numbers of PI in case they have any queries. The consent forms and assent forms were distributed to 1191 eligible parents and their children, out of which 1116 (93.7%) agreed to participate. In all, the total number of children recruited were 982 (82.5 %); 505 from school A and 477 from school B. Out of them, 496 (50.5) were boys. To improve recruitment and to maintain interest of the participants posters will be developed which will be posted in each class and in the corridors.

## Assignment of interventions

This is a non-randomized study. One school is allocated to SHEPP intervention after baseline data collection while the other school will carry on routine activity and will be subjected to intervention after follow-up data collection. The decision of selection of school for intervention therefore was based on convenience and was not randomized. The data collectors who will collect data will be blinded to the assignment of intervention. The physical activity trainer will conduct sessions in the intervention school and will have no role during the data collection.

## Data collection, management, and analysis

### Data collection

Data collection will be done by trained data collectors who will be trained by the primary investigator for (a) filling data collection forms, (b) measurement of blood pressure, waist circumference, height, and weight. Assessment of outcomes will be done at baseline and then after 10 months of intervention by the data collectors. Physical activity would be assessed using the validated youth physical activity questionnaire modified for children at baseline and follow-up [36]. Based on the findings of a previous pretesting and piloting study (unpublished), netball, rugby, skiing, snowboarding, sledging, skateboarding, and walk-the-dogs were excluded, as these are very uncommon activities and sports in our settings. However, some common traditional games such as Kabaddi (wrestling), Pahel dooj (hopscotch), Kho kho (game of chase), Pithu garam (dodge ball), kittening, bay blades, marbles, and activities related to ascending stairs were included. In addition, religious activities like performing prayer, recitation of Quran—the holy book, and household chores like sweeping, moping, cooking, dishwashing, and grocery purchase were incorporated. Finally, the modified version consists of 71 different activities in the same seven domains of the original YPAQ form. Physical activity will also be measured objectively using Mi wrist bands for at least 5% (*n* = 50) of the participants, to check the validity of physical activity measured by the questionnaire. Mi wrist band has shown 96% accuracy of measuring physical activity when compared to the gold standard [37]. Dietary assessment will be done using a 24-h dietary recall. Number of raw fruit and vegetables, number of sugar-sweetened beverages and snacks/day that are eaten will be recorded at baseline and follow-up. The dietary recalls will be conducted by trained staff on non-consecutive day. Blood pressure will be recorded using Omron m5 monitors using a pediatric cuff [38].. Weight would be recorded using Tanita’s digital weight scales. Community-setting aluminum scale was used to measure height with subjects standing barefooted (without head cover).

### Data management

The questionnaires would be checked there and then by data editors who will edit data collection forms. Each data set will be edited twice. Any missing data would be communicated to the data collectors for rechecking. The data would be entered by two independent data entry operators separately in Epi data version 3.0. The data cleaning would be done by merging the two data and any discrepancy between the two records would be looked and corrected from the case report forms. The final (cleaned) data would be logically checked and transformed into SPSS version 21 for the analysis.

#### Statistical analysis

Mean (SD) would be used for quantitative variables and frequency and percentage for categorical variables. Recruitment, retention, and treatment fidelity will be reported as percentage. Student *t* test would be used to compare quantitative variables (change in physical activity, BP, BMI, and waist circumference before and after intervention) between groups. Chi-square test to compare qualitative variables like percent increase in fruits and vegetables between the 2 groups from baseline to follow-up. Analysis would be done on intention to treat basis, that is, all participants will be analyzed in the same group as at time of allocation.

## Methods: monitoring

Data monitoring will be done by random checks on the field site while data is being collected by the research staff. Additionally, a logbook will be maintained to log all the healthy heart educational sessions and physical activity sessions.

### Adverse events

During the exercise, any injury, vasovagal episodes will be noted down by the physical trainer and would be informed to the PI and the participant will be provided first aid in the school clinic. If needed the participant will be referred to a local area hospital and will be accompanied by the research staff to the hospital. Any cost will be borne by the investigators. If participants were unable to attend 3 sessions consecutively then they will be considered non-adherent. This will be noted in the logbook. Any participant who discontinues participating in the intervention will be considered as drop out.

## Ethics and dissemination

Ethical approval from the ethics review committee, Aga Khan University has been obtained (ERC number 2571-Med-ERC-13). Informed consent and assent have been taken from participants by the research staff. Confidentiality will be maintained while data is being collected by removing the identifiers.

## Discussion

Dietary behaviors, physical activity, and sedentary lifestyle are independent predictors of overweight and higher BMI among Pakistani primary school children [39]. From our previous non-randomized pilot study, we showed that such a physical activity program is feasible in girls’ schools and potentially beneficial to their BMI and BP [30]. The importance of the current study is that it includes both parents and teachers, who are of prime importance in the behavior adopted by the children. Furthermore, an interactive video and activity-based health education session is also incorporated in the SHEPP. Teacher training may be a key element for successful interventions. To overcome time constraints, a suggested solution is to integrate physical activity into daily routines and other areas of the preschool curriculum [40]. Additionally, parental barriers are associated with the time that children spend in both active and sedentary pursuits [41].

### Limitations

The limitation of this study is the lack of randomization, including a select group of schools. This might result in selection bias, thus limiting the internal validity of the study. Secondly, the objective measurement of physical activity through an electronic wrist band is done only for 5% of the population; however, using this on the entire population would not have been cost-effective. We have not used direct objective measures to test the knowledge of children before and after the intervention; however, we will measure indirectly the change in behavior by looking at their physical activity and dietary behaviors. Also, testing them on knowledge in addition to regular schoolwork might have reduced their interest in SHEPP. Another important limitation is that we could not convince the school authorities to incorporate the physical activity sessions 4 times a week due to lack of time slots during school time. However, we will try to compensate for this by introducing between class activity and physical activity in assembly. The Pax good behavior game will be implemented in a basic format and might not be implemented in an ideal setting due to lack of training resources.

## Conclusion

SHEPP is a unique health education program for children as it focuses on children while involving the parents and teachers into the behavior change process, if found feasible with potential positive effects on physical activity and dietary behaviors, we will replicate this on a large scale implementation study in public sector schools also.

## Data Availability

Data sharing does not apply to this article as no datasets were generated or analyzed during the current study.
